# Proceedings of the Ohio Medical Convention Held at Columbus, May 12, 1846

**Published:** 1846-08

**Authors:** 


Art. VI—*Proceedings of the Ohio Medical Convention held at Columbus, May* 12, 1846. Dayton: R. N. & W. F. Comly. 1846.

We have received a copy of the Proceedings of the Medical Convention of Ohio, in which we find a good share of solid medical matter. The Convention is well sustained, having the countenance and support of the best medical talent in the State, and is very manifestly exerting a happy influence upon the profession in that quarter. Among the doings of the Convention, we perceive that it has appointed delegates to the National Medical Convention to be held at Philadelphia next spring. An excellent code of *medical ethics* is printed in connexion with the medical reports in this pamphlet, and as it has a prominent place among the proceedings of the Convention, it is to be hoped it will be diligently pondered and faithfully observed by its members.

The first medical report is in relation to Typhoid fever, of which the committee appointed to investigate the subject find it difficult to give a satisfactory account, for the reason that they have not been able to collect any facts respecting the post-mortem appearances exhibited by the disease in Ohio. The report says:

"It cannot be doubted, that the character of the fevers of the West is gradually undergoing a change. The intermittent and remittent forms of disease, are gradually being replaced by those more continued. This change is more observable and marked in some localities than others, and probably depends in a great degree, if not entirely, upon the clearing, draining, and cultivation of the soil. In numerous places known to us, where the malarious diseases were formerly very prevalent, they are now scarcely known; and in a number of instances quite extensive epidemics have prevailed, agreeing in their main symptoms with what is described as typhoid fever."

The next report is of a case of deformity resulting from a burn, in which an operation was successfully performed by Dr. Thompson, Surgeon General of Ohio:

"This case was presented to the Convention by drawings, representing a young lady 20 years old, very much deformed by a burn which occurred when she was a child of 4 years, and the relief afforded by an operation.

"The burn occurred on the breast, neck, and chin. The chin was drawn down to the sternum, the mouth open, and the head permanently bent forward.

"An incision was made into the modulated tissue, about midway of the sternum; the integuments dissected up to the lower maxillary bone; the underlying cellulo-fibrous tissue of this point divided, to admit a proper elevation of the head, and fashion a chin. The next step in the operation was to adujust the integuments to the parts thus prepared, and secure them by appropriate bandages.

"First dressing, patent lint—subsequent, various. Elastic retaining bandages were used during the entire healing process. The flap, in its main extent, united by the first intention.

"The operation was entirely successful—the chin and head resumed their natural position, and a fine, intelligent young lady, who had had an unsightly deformity for sixteen years, was made of a very respectable appearance. Her general health, too, which had become very poor before the operation, was completely restored."

This is followed by an essay on *obstetric auscultation*, by Professor Butterfield, of Willoughby Medical College. Notes of a paper on version of the uterus, and of one on animal magnetism are given, but the articles have not been published. A case of "*Spontaneous dry Gangrene*," attended with hydrophobic symptoms, is reported by Dr. R. Hills, at length. The subject was a lady 41 years of age, who had suffered from a severe attack of pneumonia, for which she was actively treated. After two or three relapses, in which the lancet was found necessary, and after exposure to a cold room in waiting upon a sick daughter, she was seized with a chill, which was followed by acute pain in one of her lower extremities. Dr. Hills found the limb pale, "of a cadaverous appearance below the knee, and somewhat shrunken in its volume." Five days afterwards signs of mortification appeared; the cuticle separated over the lower part of the leg and foot which had become slightly swollen.

"On April 4th, the toes and lower third of the foot assumed a *tawny hue*, or brown scorched color, and during the succeeding three or four days, this hue increased and the parts became shrivelled, hard, horny, and semi-transparent. The same changes soon became apparent on the side of the leg spoken of as being white and corrugated. At the same time the remaining portion of the leg became more tumefied, of a greenish black color, and very fetid. In the mean time, we wrapt a blister entirely around the limb above the knee, applied the fermenting, charcoal and linseed poultices, &c., to the knee itself, and the upper part of the leg, and encased the lower portion in charcoal alone, for the purpose, simply, of removing the fetor. She gradually sank, however, until the 7th of April, when at noon her pulse was 150 and very feeble, and by evening it was so indistinct that it could not be counted. It remained so during the night and the whole of the 8th. Her respiration at the same time was irregular, deglutition difficult, and voice almost inarticulate. Quinine and brandy were freely administered while in this condition, and in the evening of the 8th she revived, and the pulse became distinct, and when first counted was 140. By the morning of the 9th, it was 120, and she had correspondingly improved in other respects. In this condition she remained until the 14th, six days, the sphacelation slowly proceeding up the limb, at the rate of three or four lines per day, preceded by a band of purple redness, with slight œdema."

In ten days from this time the sphacelation ceased to progress, "and a line of demarcation began to form below the knee, first indicated by the crimson band changing to a scarlet color." The patient had sunk so low that her physicians were deterred from amputation in the ordinary way, but a separation of the putrid limb was determined upon and was effected through the dead parts, the patient being unconscious of the operation until she heard the grating of the saw against the bone. Two days after this operation, the patient's condition appeared improved, and hopes of her recovery began to be entertained; but symptoms of hydrophobia came on five days subsequently, which terminated her sufferings on the fourth day after their occurrence, and forty-five days "from the first invasion of disease." These symptoms are thus described by Dr. Hills:

"When drink was brought to her, as she was about to take it, there was a general nervous disturbance, amounting to a shudder, with twitchings of the muscles of the face, neck and arms, though she would succeed in taking her drink. These difficulties increased during the afternoon until 7 P. M., when I first became aware of it. The convulsive movement, or *strong, clonic spasms* of the muscles of the face, neck, arms, and chest, increased by attempts to drink, seemed now to arrive at their acme, and when I came into her presence, the eye-balls were thrown convulsively upwards, the eyes open and staring, the muscles of the face horribly distorted, respiration nearly suspended, having been, immediately preceding, that of a person walking into cold water. This condition lasted about half a minute. At intervals of from one to five or ten seconds, there were short, sharp and strong jerks of various muscles, principally confined to the upper portion of the body, still continuing. In ten or fifteen minutes, on attempting to give her an anti-spasmodic, she was thrown into the condition described as when I first saw her, which lasted about the same length of time, a half minute. These paroxysms, so to speak, of convulsions, during the next four hours, were as frequent as once in 20 or 30 minutes, the short, sharp jerks continuing in the intervals with greater or less frequency. The paroxysms were sometimes brought on by attempts to drink, by bodily exertions, and sometimes without an apparent immediate cause. From 8 to 10 o'clock she took no drink, all attempts being unsuccessful, though various means were tried. We could not even get ice into her mouth, though there was no *trismus.*

"She was frequently complaining of her inability to drink, as her thirst was great. At an early part of the evoning, a warm bath was suggested, with which suggestion she seemed pleased, but when it was ready for her use, the mere information that it was ready, produced such shuddering and convulsive movements, with dread and reluctance in her mind, that we were compelled to desist. Her head, upon the upper portion of it, was exceedingly hot, for which we kept ice constantly applied in a bladder. For the first few hours, the functions of the brain seemed unaffected, but then the mind became slightly wandering, confused for a few moments, then clear again; but upon the whole the confusion increasing. In the latter part of the night, there was an abatement of the troubles. She was still convulsed, but could drink with tolerable ease, though with agitation in the effort. Her pulse had during the night been small, irregular, and rapid, the extremities cool. In the morning of April 28th, some general reaction took place, or the pulse was fuller and the extremities warmer. At 12, M., the convlsive actions again increased, and were frequent and severe until 3 or 4, P. M., when they again diminished, the powers of the system evidently giving way—the delirium having become constant, and the pulse very small and irregular, her muscular strength diminished, &c., and by midnight, deglutition was difficult, and her voice became inarticulate. There was constant watchfulness, with delirium, and after lingering until 9, P. M., of the 30th, death relieved her from her varied and greatly protracted suffering, 45 days from the first invasion of disease.

"Since her death, information was given by an aged and intimate female acquaintance of the family, that when Mrs. L. was from 14 to 16 years of age, from 25 to 27 years since, she was bitten by a favorite house dog of her own, while it was laboring under some species of fits, with which it died, or for which it was killed, she thinks the latter.

"Of these facts the immediate family have no recollection, except that they possessed such a dog.

"This case, it appears to me, calls up several questions of great interest, among which are the following:

"Was the gangrene *spontaneous*, or the sequela of the previous disease?

"Was the convulsive condition at the close, truly hydrophobia, or traumatic tetanus? If the former, it is remarkable as to the time that the virus remained inactive; if the latter, it is equally so as to its simulating hydrophobia."

We should not be disposed to attach much importance to the story of the "aged female," that the patient, when a girl, twenty-seven years before, "was bitten by a favorite house-dog of her own." The irritation of the sphacelated limb strikes us as a cause quite sufficient to have elicited all the symptoms in the case.

Reports of several surgical operations by Dr. Howard make up the remainder of this pamphlet. One of these, the removal of a large urinary calculus from a female, possesses a good deal of interest. By means of an instrument contrived by the operator, Dr. H. was able to dilate the urethra to such an extent, that but little cutting was necessary. Dilatation by sponge tents and bougies had been found impracticable on account of the irritability of the parts. The operation is thus described:

"The patient being placed as in the lateral operation for the male, without any difficulty, I introduced my dilator, and readily separated the arms of the instrument by means of the thumb-screw, so that in fifteen minutes I was enabled with very little pain to the patient, to place my finger upon the stone, while the instrument still remained in the urethra. As it was not safe to depend upon dilatation farther, I introduced the bistoury and divided the urethra upward and outward, after Liston's plan. This division increased the facility of dilatation to almost any extent. I could now feel the stone in every direction. I immediately introduced a medium sized forceps, but found the stone too large to escape with impunity to my patient. I again introduced the dilator, distended the parts, and then made another incision outward and upward on the opposite side. This enabled me to command the stone, and to remove it without much force."

In a week, the patient was able to retain her urine from two to four hours, and ultimately regained the power completely.

